# A Combined Nomogram Model to Predict Disease-free Survival in Triple-Negative Breast Cancer Patients With Neoadjuvant Chemotherapy

**DOI:** 10.3389/fgene.2021.783513

**Published:** 2021-11-12

**Authors:** Bingqing Xia, He Wang, Zhe Wang, Zhaoxia Qian, Qin Xiao, Yin Liu, Zhimin Shao, Shuling Zhou, Weimin Chai, Chao You, Yajia Gu

**Affiliations:** ^1^ International Peace Maternity and Child Health Hospital, Shanghai, China; ^2^ Shanghai Cancer Center, Fudan University, Shanghai, China; ^3^ Institute of Science and Technology for Brain-Inspired Intelligence, Fudan University, Shanghai, China; ^4^ Shanghai United Imaging Medical Technology Co., Ltd., Shanghai, China; ^5^ Ruijin Hospital, School of Medicine, Shanghai Jiao Tong University, Shanghai, China

**Keywords:** radiomics, neoadjuvant chemotherapy, nomogram, triple-negative breast cancer, disease-free survival

## Abstract

**Background:** To investigate whether the radiomics signature (Rad-score) of DCE-MRI images obtained in triple-negative breast cancer (TNBC) patients before neoadjuvant chemotherapy (NAC) is associated with disease-free survival (DFS). Develop and validate an intuitive nomogram based on radiomics signatures, MRI findings, and clinicopathological variables to predict DFS.

**Methods:** Patients (*n* = 150) from two hospitals who received NAC from August 2011 to May 2017 were diagnosed with TNBC by pathological biopsy, and follow-up through May 2020 was retrospectively analysed. Patients from one hospital (*n* = 109) were used as the training group, and patients from the other hospital (*n* = 41) were used as the validation group. ROIs were drawn on 1.5 T MRI T1W enhancement images of the whole volume of the tumour obtained with a 3D slicer. Radiomics signatures predicting DFS were identified, optimal cut-off value for Rad-score was determined, and the associations between DFS and radiomics signatures, MRI findings, and clinicopathological variables were analysed. A nomogram was developed and validated for individualized DFS estimation.

**Results:** The median follow-up time was 53.5 months, and 45 of 150 (30.0%) patients experienced recurrence and metastasis. The optimum cut-off value of the Rad-score was 0.2528, which stratified patients into high- and low-risk groups for DFS in the training group (*p*<0.001) and was validated in the external validation group. Multivariate analysis identified three independent indicators: multifocal/centric disease status, pCR status, and Rad-score. A nomogram based on these factors showed discriminatory ability, the C-index of the model was 0.834 (95% CI, 0.761–0.907) and 0.868 (95% CI, 0.787–949) in the training and the validation groups, respectively, which is better than clinicoradiological nomogram(training group: C-index = 0.726, 95% CI = 0.709–0.743; validation group: C-index = 0.774,95% CI = 0.743–0.805).

**Conclusion:** The Rad-score derived from preoperative MRI features is an independent biomarker for DFS prediction in patients with TNBC to NAC, and the combined radiomics nomogram improved individualized DFS estimation.

## Introduction

Triple-negative breast cancer (TNBC) is a clinical challenge because of its invasive nature, high risk of distant metastasis, and poor prognosis. Compared with other breast cancer patients, TNBC patients are 2–3.5 times more likely to have distant recurrence (Fatayer et al., 2016). It has been demonstrated that the probability of a pathological complete response (pCR) is higher in TNBC patients who receive neoadjuvant therapy (NAC) (close to 31% at present) than in patients with other molecular subtypes, suggesting that NAC improves DFS in this group of patients (Houssami et al., 2012). However, pCR alone is not enough to predict the long-term recurrence-free survival rate of patients with TNBC, and an efficient prognostic biomarker is urgently needed to help stratify patients and create treatment guidelines.

Recently, some studies have indicated that radiomics can be used to obtain a series of related parameters to quantify the heterogeneity of lesions and shows promise for improving tumour prognosis. In previous studies, the radiomics nomogram provided a promising prediction of neoadjuvant chemotherapy efficacy in breast cancer patients based on pretreatment MRI images (Bian et al., 2020; Chen et al., 2020). Another study reported that the radiomics signature(Rad-score) could be used for DFS prediction in HER-2-positive invasive breast cancer treated with NAC, and the radiomics-clinicoradiologic-based nomogram may potentially be useful for personalized treatment strategies (Li et al., 2020). However, there is no relevant research on TNBC.

Dynamic contrast-enhanced magnetic resonance imaging (DCE-MRI) has excellent sensitivity and good specificity for breast cancer diagnosis and plays an important role in characterizing the heterogeneity of tumours. Most studies involving radiomics analysis only use the initial enhancement phase of DCE-MRI, and the additional value of radiomics calculated from later enhancement images was limited. Nevertheless, the radiomics features derived from the phases of multiple DCE-MRI images cannot be ignored, which may imply more information changing over time points.

The purpose of this study was to investigate whether the radiomics derived from all DCE-MRI phases obtained in TNBC patients before NAC are associated with DFS and to compare the combined radiomics nomogram and the clinicoradiological nomogram for their abilities in predicting DFS in patients with TNBC treated with NAC.

## Materials and Methods

The institutional review board approved this two-institution study and retrospective radiomics data analysis (approval No: 2004216-14), and the requirement for written informed consent was waived.

### Patients

Between August 2011 and May 2017, a total of 150 patients from two hospitals were enrolled according to the inclusion criteria. The inclusion criteria included 1) oestrogen receptor (ER), progesterone receptor (PR) and human epidermal growth factor receptor 2 (HER2) were all negative according to a core-needle biopsy performed before treatment (the HER2 score (2+) obtained based on immunohistochemistry and gene amplification was confirmed with fluorescence *in situ* hybridization), 2) patients who received NAC and underwent a final surgery, and 3) patients who underwent an examination using the same machine (Aurora Dedicated Breast MRI System, USA, Aurora). The exclusion criteria included the following: 1) patients who did not undergo a magnetic resonance examination before treatment, 2) patients whose lesions were hardly identified on breast MR images, 3) patients with confirmed systemic metastasis, 4) patients with no final pathological results after treatment, and 5) patients who were lost to follow-up after operations. Finally, all patients were required to undergo an MR examination within 30 days before neoadjuvant therapy. The following information was also recorded for all patients: age, menopausal status, start date of NAC, clinical stage, pre-NAC-T stage and N stage, tumour histologic type, Ki67, surgery type, and date of progression (local recurrence and distant metastasis) to determine duration (months) of DFS. DFS was calculated from the date of surgery to the date of breast cancer recurrence and metastasis, the last confirmation of no evidence of disease, or the most recent follow-up examination.

### Magnetic Resonance Imaging

Before treatment, all MR scans were performed with an AURORA 1.5T breast magnetic resonance machine (Aurora Dedicated Breast MRI System, United States, Aurora). The patients underwent this procedure in the prone position with both breasts naturally suspended in a dedicated breast coil. The scanning range included the bilateral breasts and axillary regions. DCE-MRI was performed using axial T1-weighted fat suppression (TE/TR = 5 ms/29 ms, slice thickness = 1.5 mm with no gap, FOV = 360 mm, matrix = 360 × 360) and consisted of one precontrast and three consecutive postcontrast dynamic series. Gd-DTPA was injected into the dorsal hand vein via a bolus injection (0.1 mmol/kg) at a rate of 2.0 ml/s. The scanning time for each phase was approximately 2 min.

All medical images and clinical records were independently reviewed by two radiologists specializing in breast imaging diagnosis (with 5 and 15 years of experience, respectively). The morphologic manifestations (such as mass or nonmass enhancement and TIC curve) of each lesion were determined according to the 2013 Breast Imaging Reporting and Data System (BI-RADS) MR imaging lexicon standard proposed by the American College of Radiology.

### Tumour Masking and Inter-Observer Reproducibility Evaluation

**FIGURE 1:**
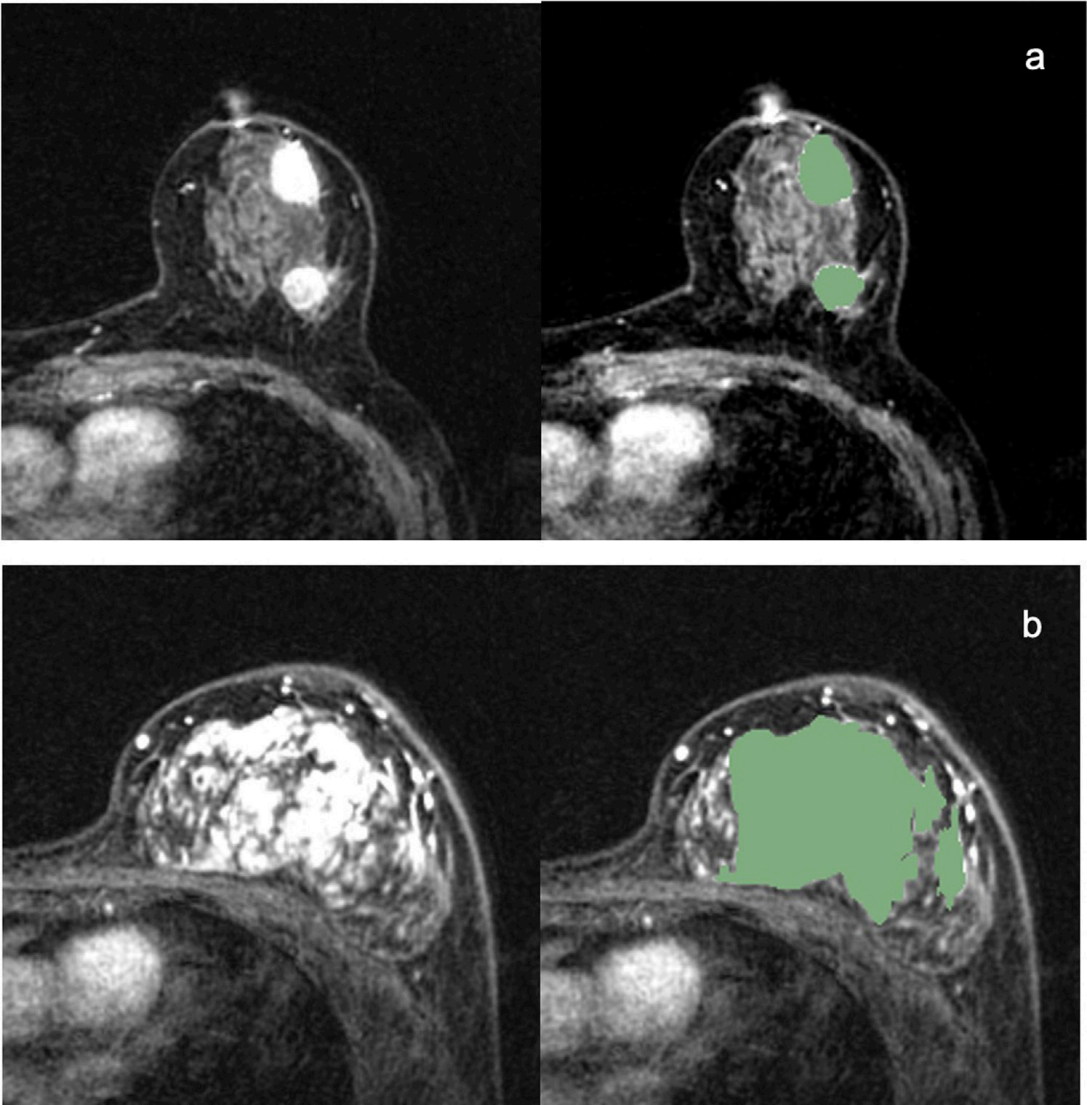
Examples of 3D segmentations of Triple-negative breast cancers.

ROIs were manually drawn by the radiologist on the whole volume of the tumours (including the necrotic regions) with 3D Slicer software (https://www.slicer.org). The 3D segmentation ROIs of the whole tumour were first created on the first post-contrast DCE images and then propagated to the pre-contrast and the other two post-contrast series of DCE images. For multifocal/centric and nonmass enhancement tumours, ROIs were drawn over all lesions. Examples of 3D segmentation are shown in Figure 1. Figure 1A is MR images of TNBC with multifocal/centric masses. The green area represents the scope of ROI delineation, and each lesion is delineated by layers. Figure 1B is MR images of TNBC with non-mass lesions.The green area is delineated by ROI and delineated according to the scope of enhancement.

Using 50 randomly selected samples, the interobserver reproducibility of ROI detection and radiomic feature extraction was measured. Two experienced radiologists (BQX and QX) described the ROI independently, and then the radiomic features extracted from the above two ROIs were compared to obtain the interclass correlation coefficient. An ICC score greater than 0.8 was interpreted as satisfactory agreement. The ICC for the radiomic features was defined as high (ICC ≥ 0.8), medium (0.8 > ICC ≥ 0.5) or low (ICC < 0.5).

### Treatment Regimen and Criteria for pCR and Recurrence

All patients received paclitaxel sequential/combined anthracycline neoadjuvant chemotherapy with or without platinum. The median duration of NAC was 4 (range, 4–8) months. pCR was defined as ypT0/is and ypN0, which indicate the absence of residual invasive carcinoma in breast tissues with or without ductal carcinoma *in situ* and the absence of any residual cancer in the sampled axillary lymph nodes. A pathological response was determined by senior breast pathologists. Recurrence was defined as local-regional (confined to the ipsilateral breast or chest wall and/or axillary, infraclavicular or supraclavicular lymph nodes) and distant metastasis (to other parts of the body or the contralateral breast). Breast cancer recurrence was confirmed by biopsy, and metastasis was confirmed by biopsy when appropriate or on the basis of an imaging assessment, including PET/CT and other imaging modalities.

### Radiomics Analysis, Feature Selection and Rad-Score

The radiomics signature included 1316 radiomics features that were extracted from the training group by the PyRadiomics package in Python software (v. 3.6, Python Software Foundation, https://www.python.org/). All these features were classified into 3 groups (Table 1). To characterize the textural changes observed on DCE images over time series, we measured ten new sequential features for each texture feature described in group b (Supplementary Table S1). All these features have been applied in previous radiomics studies (Li et al., 2020). Forward stepwise regression was applied to select features. Rad-score was calculated for each patient via a linear combination of selected features that were weighted by their respective coefficients. Feature selection was achieved using the Statistics Toolbox in MATLAB (v. R2018a; MathWorks, Natick, MA).

**TABLE 1:** Three groups of extracted features.

	Group	Number (features)	Description
a	Shape features on DCE (DCEshape)	14	The 14 shape-based features were calculated based on the first postcontrast DCE images
b	Texture features based on DCE images with 4 time series (DCEtexture)	372	The 93 texture features (including 18 first-order features, 24 grey-level co-occurrence matrix (GLCM) features, 16 grey-level run length matrix (GLRLM) features, 16 grey-level size zone matrix (GLSZM) features, 5 neighbouring grey tone difference matrix (NGTDM) features, and 14 grey-level dependence matrix (GLDM) features) were calculated based on these four series image sets to yield 372 features
c	Sequential features based on DCE images (DCEsequential)	930	The first six features, including mean, variance, kurtosis, skewness, energy, and entropy, were extracted for each individual subject. The other four features, including Kendall-tau-b, conservation, stability, and dispersion, were calculated for the interactive information between the current subject and the remainder of the subjects. Therefore, a total of 930 DCEsequential features were extracted from 93 texture features

## Statistical Analysis

We compared patient characteristics using commercially available statistical software (IBM SPSS 24.0). When appropriate, significant differences between the training and validation groups were assessed by the Chi-square test, Fisher’s test or *t*-test. A two-sided *p* value of less than 0.05 indicates a significant difference. The Rad-scores were divided into two groups (high-risk vs low-risk) using receiver operating characteristic (ROC) curve analysis according to optimal cut-off value determined by maximizing the Youden index (sensitivity + specificity-1). Significant variables in the univariate Cox proportional hazard model (*p* < 0.05) were included in the multivariate analysis. The combined radiomics nomogram incorporated the radiomics signature and various independent risk factors based on multivariate analysis in the training group and was then validated in the validation group. The predictive ability and discriminatory performance of each established model were evaluated using an index of probability of concordance (C-index), and the C-index between the predicted probability and actual outcome was calculated to evaluate the predictive ability and discrimination of the model (Wolbers et al., 2009). The value of the C-index ranges from 0.5–1.0, with 0.5 indicating random chance and 1.0 indicating perfectly accurate discrimination. The nomograms were subjected to bootstrapping validation (1000 bootstrap resamples) to obtain a relatively corrected C-index.

## Results

### Patient Characteristics

The clinicopathological and MR imaging characteristics of the training and validation groups with TNBC are listed in Table 2. Except for the clinical stage, pre-NAC N stage and pCR status, there were no differences between the training and validation groups. The median follow-up time was 54 months (range, 1–101 months) for the training group and 48 months (range, 1–88 months) for the validation group. There were 45 (30.0%) recurrences, 30 (20.0%) in the training group and 15 (10.0%) in the validation group, including 35 patients with distant metastasis (one also had additional local-regional recurrence), 8 with local-regional recurrence only, and 2 with contralateral breast cancers.

**TABLE 2:** Comparison of clinical and pathological and pretreatment MR imaging characteristics between training and validation groups.

Characteristics	Training group (*n* = 109)	Validation group (*n* = 41)	*p*
Age, mean (SD), y	47.3 ± 11.1	48.6 ± 13.3	0.545
Menopausal status			0.322
Premenopausal	63(57.8)	20(48.8)	
Postmenopausal	46(42.2)	21(51.2)	
Clinical Stage			0.007^a^
II	83(76.1)	22(53.7)	
III	26(23.9)	19(46.3)	
Pre-NAC T-stage			0.061
T1	10(9.2)	4(9.8)	
T2	68(62.4)	16(39.0)	
T3	22(20.2)	14(34.1)	
T4	9(8.3)	7(17.1)	
Pre-NAC N-stage			0.032^a^
N0	38(34.9)	10(24.4)	
N1	55(50.5)	21(51.2)	
N2	7(6.4)	9(22.0)	
N3	9(8.3)	1(2.4)	
Pathological type			0.575
IDC	105(96.3)	41(100.0)	
ILC,IMPC	4(3.7)	0(0.0)	
KI-67			0.090
≤14%	6(5.5)	6(14.6)	
>14%	103(94.5)	35(85.4)	
Surgery type			0.075
Breast conservation	21(19.3)	3(7.3)	
Mastectomy	88(80.7)	38(92.7)	
Features at MR imaging			0.455
Mass	86(78.9)	30(73.2)	
Nonmass	23(21.1)	11(26.8)	
Kinetics			0.684
Washout	104(95.4)	38(92.7)	
Plateau or persistent	5(4.6)	3(7.3)	
Multi-focal/centric disease			0.695
Present	31(28.4)	13(31.7)	
Absent	78(71.6)	28(68.3)	
pCR			0.022^a^
Yes	46(42.2)	9(22.0)	
No	63(57.8)	32(78.0)	
Lymphovascular invasion			0.052
Present	23(21.1)	15(36.6)	
Absent	86(78.9)	26(63.4)	
Disease-free survival			0.280
Yes	79(72.5)	26(63.4)	
No	30(27.5)	15(36.6)	

Data are expressed as *n*(%) unless otherwise specified.

The *p* values for age were determined by *t* test, while other *p* values were determined by Chi square or Fisher exact tests, as appropriate.

a indicate statistical significance (*p*<0.05).

IDC, invasive ductal carcinoma; ILC, invasive lobular carcinoma; IMPC, invasive micropapillary carcinoma; *p*CR, pathological complete response.

### Radimics Analysis, Rad-Score Building and Validation

The ICC for radiomic features between the two radiologists BQX and QX ranged from 0.8732 to 0.9671. Two radiologists generally reached a consensus on the delineations. To verify the importance of the new features, two different Radimics models were delevoped. Model 1 only uses the features derived from the first postcontrast phase, while Model 2 uses the features derived from all dynamic phases, including the new features. The results for the two models are shown in Table 3. Model 2 achieved a predictive accuracy of 85.4%, sensitivity of 50.0%, specificity of 97.6%, PPV of 88.0%, and NPV of 85.0%, which was more robust than Model 1. Finally, Model 2 was selected for the following study.

**TABLE 3:** Summary of radiomics model1 and model2 results.

	Accuracy	Sensitivity	Specificity	PPV	NPV
Model1 (1st PC phase)	76.6%(74.3–78.0)	17.4%(10.7–21.4)	97.1%(95.1, 98.8)	68.1%(50.0–83.3)	77.3%(76.0–78.2)
Model2 (All phases, 1pre-contrast and 3 PC phases	85.4%(84.4–86.2)	50.0%(46.4–50.0)	97.6%(96.3–98.8)	88.0%(82.4–93.3)	85.0%(84.8–85.1)

Confidence intervals are in parenthesis. Above two models were performed using a fine Gaussian support vector machine and conducted using 5-fold cross validation to overcome overfitting. The procedure was repeated for ten rounds to average the estimates of performance.

PC, post-contrast; PPV, positive predictive value; NPV, negative predictive value.

**FIGURE 2:**
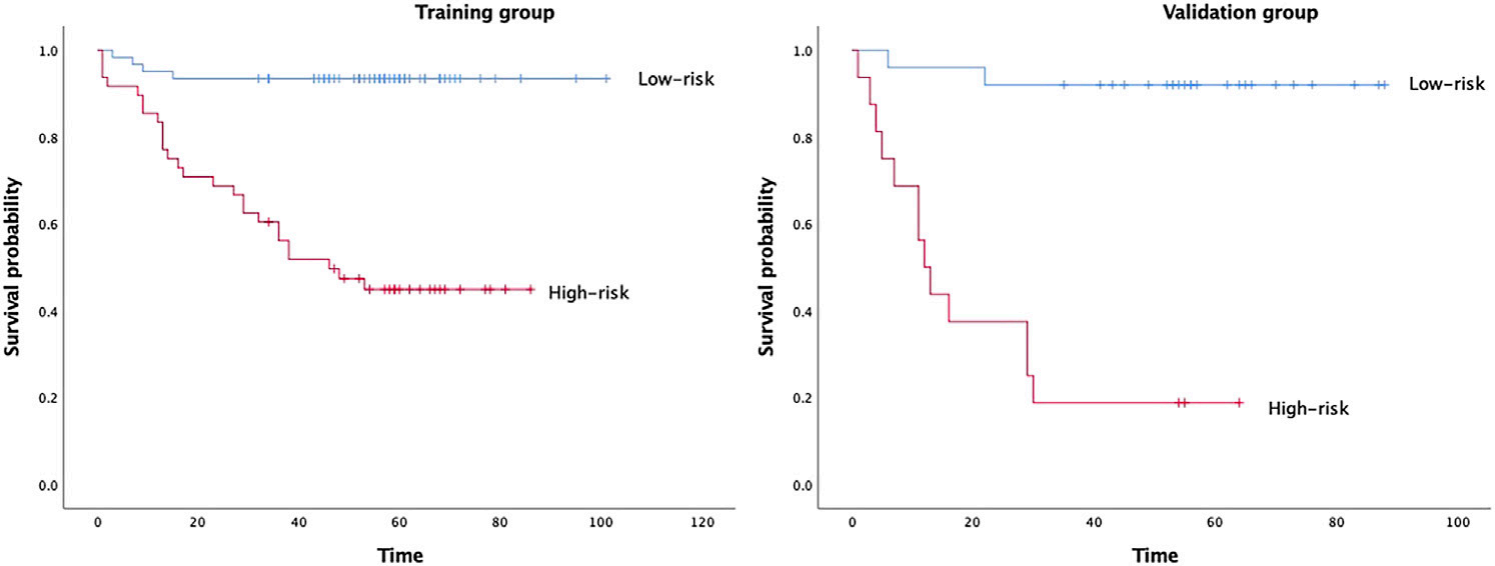
Kaplan–Meier survival analyses according to the radiomics signature with low-risk and high-risk patients in training and validation groups.

In Model 2, six textural features were selected for predicting DFS after forward stepwise regression selection, and the Rad-score calculation formula is presented:y = 0.25688+(−0.12986)×Skewness_glcm_Imc1+(−0.13965)×Entropy_firstorder_RootMeanSquared+(−0.094626)×Entropy_ngtdm_Busyness+0.10472×Kendall-tau-b_glcm_Idmn+(−0.23802)×Conservation_glcm_DifferenceAverage+0.2713×Conservation_ngtdm_Complexity. The above selected features are all from group c (DCEsequential). There was a significant difference in Rad-scores between the recurrence and no recurrence groups (*p*<0.001) in the training group. The median Rad-score was 0.2349 (range, −0.3165 to 0.9846; interquartile range, 0.1038–0.3812). The optimum cut-off value generated by the ROC curve was 0.2528, and the AUC was 0.852 (95% CI, 0.773–0.932). Using this threshold value, patients were classified into a high-risk group (Rad-score ≥ 0.2528) and a low-risk group (Rad-score < 0.2528). Kaplan-Meier curves showed that the radiomics signature was associated with DFS in the training group (*p* < 0.001), and this finding was confirmed in the validation group (*p* < 0.001) (Figure 2).

### Univariate and Multivariate Analyses of the Risk Factors for RFS

The results of the univariate and multivariate analyses of the risk factors for RFS in the training group are shown in Table 4. A higher Rad-score, multifocal/centric lesions, nonmass lesions, ILC/MIPC histological type, non-pCR and lymphovascular invasion were associated with worse DFS. Furthermore, in the multivariate Cox analysis, a higher Rad-score (DFS odds ratio 26.685; 95% CI 6.654–107.010; *p* = 0.000), multifocal/centric lesions (DFS odds ratio, 2.522; 95% CI, 1.160–5.481; *p* = 0.020), and pCR status (DFS odds ratio, 0.285; 95% CI, 0.100–0.810; *p* = 0.019) remained independent prognostic factors (Table 4).

**TABLE 4:** Univariate and multivariate analysis of disease-free survival in training group.

Characteristics	Univariate analysis			Multivariate analysis		
	OR	95% CI	*p* value	OR	95% CI	*p* value
Age,<35 years versus ≥35 years	1.775	0.538–5.855	0.346			
Menopausal status, premenopausal versus postmenopausal	1.661	0.810–3.404	0.166			
Clinical Stage, II versus III	1.529	0.700–3.340	0.287			
Pre-NAC Tstage(T1 reference)			0.306			
T2	3	0.354–25.439	0.314			
T3	5.143	0.547–48.365	0.152			
T4	7.2	0.622–83.342	0.114			
Pre-NAC Nstage(N0 reference)			0.248			
N1	1.322	0.512–3.41	0.564			
N2	4.296	0.806–22.9	0.088			
N3	0.403	0.044–3.669	0.42			
Pathologic type, IDC versus ILC, IMPC	5.330	1.602–17.735	0.006^a^	0.851	0.210–3.445	0.821
KI-67, ≤20% versus >20%	0.452	0.137–1.493	0.193			
Surgery type, Breast conservation versus Mastectomy	2.252	0.683–7.426	0.182			
Features at MR imaging, Mass versus Nonmass	2.454	1.145–5.262	0.021^a^	1.565	0.639–3.832	0.327
Kinetics, Washout versus Plateau or persistent	0.659	0.090–4.84	0.682			
Multi-focal/centric disease, Present versus Absent	3.177	1.549–6.517	0.002^a^	2.522	1.160–5.481	0.020^a^
pCR, Yes versus No	0.232	0.089–0.608	0.003^a^	0.285	0.100–0.810	0.019^a^
Lymphovascular invasion, Present versus Absent	2.254	1.054–4.820	0.036^a^	0.995	0.402–2.461	0.991
Rad-score	52.829	14.821–188.300	0.000^a^	26.685	6.654–107.010	0.000^a^

OR, odds ratio; CI, confidence interval; pCR, pathological complete response.

a indicate statistical significance (*p* ≤ 0.05).

### Radiomics Nomogram Building and Validation

**FIGURE 3:**
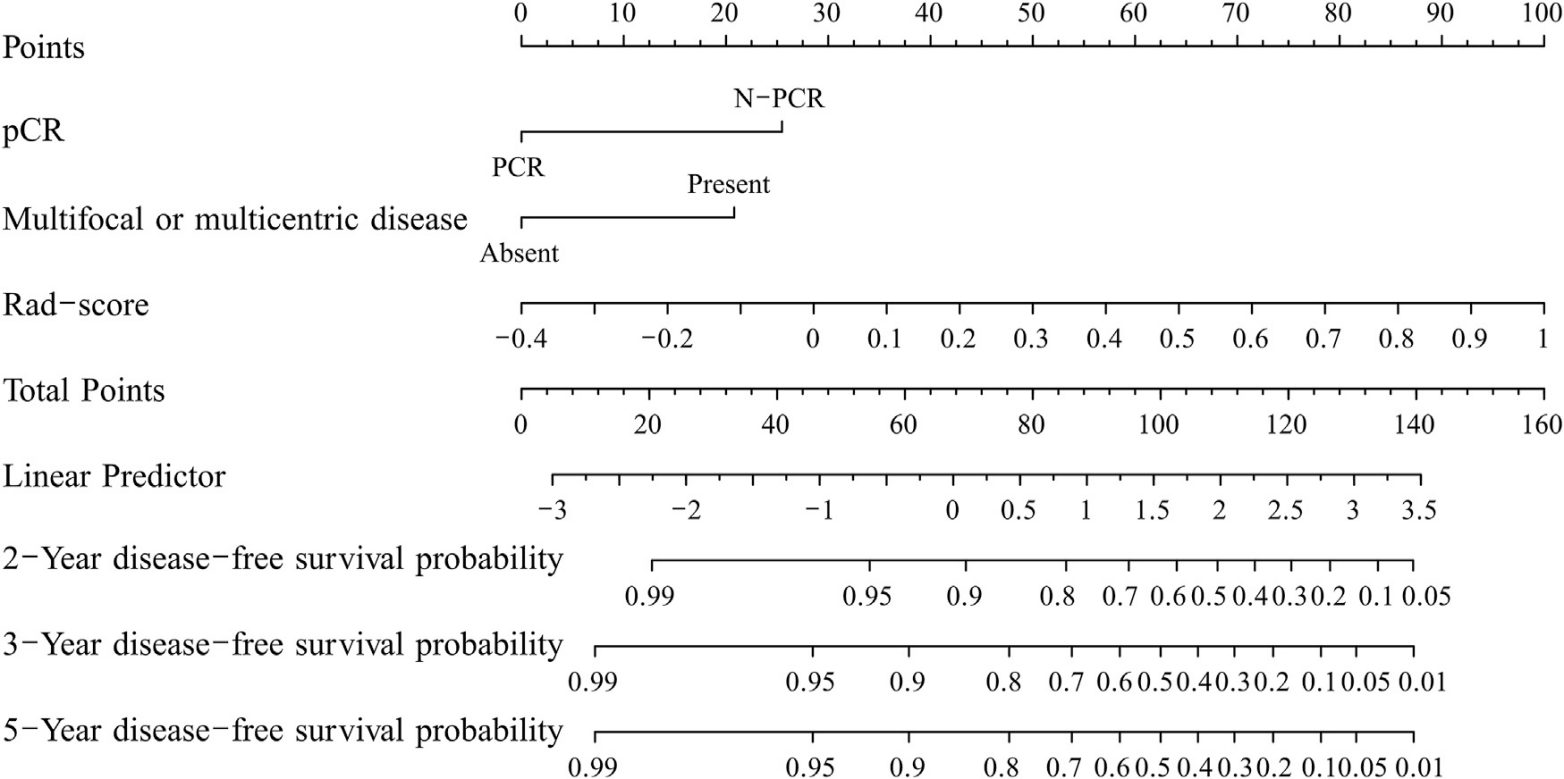
The developed nomogram for predicting disease-free survival in triple-negative breast cancer patients after neoadjuvant chemotherapy.

The C-index of the two kinds of nomogram models for the prediction of DFS in the training group and validation group is shown in Table 5. A combined radiomics nomogram was developed based on multifocal/centric disease status, pCR status, and Rad-score to predict the DFS rate for NAC among TNBC patients (Figure 3). A total score was obtained by adding each single score to estimate the 2-/3-/5-years DFS probability. The C-index was 0.834 (95% CI, 0.761–0.907) and 0.868 (95% CI, 0.787–0.949) in the training and validation groups, respectively, indicating that the combined radiomics nomogram had better discriminatory capability than the clinicoradiological nomogram.

**TABLE 5:** Performance of the two nomogram for prediction of disease-free survival.

Nomogram	Training		Validation	
	C-index	95%CI	C-index	95%CI
Combined Radiomoics nomogram	0.834	0.761–0.907	0.868	0.787–0.949
Clinicoradiological nomogram	0.726	0.709–0.743	0.774	0.743–0.805

C-index,index of probability of concordance; CI, confidence interval.

## Discussion

In our study, we demonstrated the prognostic value of multiphase CE-MRI radiomics features for patients with TNBC treated with NAC. In addition, we developed a combined radiomics model that incorporates the radiomics signature and MRI and pathology findings for the individualized prediction of DFS in TNBC patients who underwent NAC. Compared with the clinicoradiological nomogram, the combined radiomics nomogram had superior prognostic performance in DFS estimation.

For feature extraction and selection, we measured ten new sequential features to characterize the textural changes observed on DCE images over time series. These features have not previously been used or described in the domain of breast radiomics except in Li et al. (2020) and Xie et al. (2019) studies, who used new features to differentiate different subtypes of breast cancer and predict DFS in patients with HER2-positive breast cancer treated with NAC. We compared two models to investigate whether the accuracy of the radiomics model was significantly improved after adding new features. Roberto et al. (Lo Gullo et al., 2020) and Gibbs et al. (2019) both demonstrated that delayed postcontrast phases did not add any significant discriminative value to the analysis, which is inconsistent with our research results. The reason may be that we added new sequential features, but they did not include them, and the subjects of their study were subcentimetre masses that were much smaller than ours lesions. Furthermore, the sequential texture features derived from dynamic phases may capture information on both spatial heterogeneity and tumour perfusion, which is more valuable in predicting DFS than differentiating benign and malignant lesions.

In our study, the final Rad-score calculation formula included six potential features all from the new sequential features. The six selected radiomics features comprised one from skewness, two from entropy, one from Kendall-tau-b and two from conservation. Among them, other studies have also emphasized the importance of skewness and entropy in reflecting the heterogeneity of tumours. Kendall-tau-b and conservation were calculated from interactive information between the current subject and the remainder of the subjects, which means that if the changes increased, the Rad-score increased, indicating a worse prognosis. One possible interpretation is that this change may be related to the high perfusion of the tumours, and tumours with abundant blood supply tend to be more heterogeneous and have a worse prognosis. Attentionally, three of the six selected features were GLCM (grey level cooccurrence matrix), and two were NGTDM (neighbourhood grey-tone difference matrix). At present, GLCM is the most widely used texture extraction method, which has also been confirmed in assessing tumour heterogeneity and plays a very important role in various fields. The basic principle of the GLCM is based on spatial correlation between neighbouring pixels. NGTDM represents contrast, which is determined by changes in intensity between a target voxel and the surrounding neighbours and then enables the calculation of the apparent difference between neighbouring regions of voxel intensities. Contrast is also related to tumour heterogeneity; tumours with poor prognosis tend to have higher contrast (Sun and Wee, 1983). Our results also showed that the Rad-score had a promising high value for predicting DFS, which was confirmed by Kaplan–Meier survival curves in the training group (*p* < 0.0001) and in the validation group (*p* < 0.0001). Interestingly, the cut-off value (Rad-score = 0.2528. for predicting DFS was similar to QL’s study (Rad-score = 0.2523), regardless of TNBC or HER2-positive breast cancer with NAC.

There were differences in clinical stage, pre-NAC N stage and pCR status between the training and validation groups, which might be associated with differences in study populations with different hospitals. In the validation group, the later the clinical stage, the more difficult it was to achieve pCR. Various previous studies have confirmed that a tumour’s response to neoadjuvant therapy provides prognostic information. The attainment of a pCR after NAC and surgical resection improved the DFS rate of patients (Houssami et al., 2012), (Cortazar et al., 2014; Chen et al., 2017; Symmans et al., 2017), consistent with our study. However, 42.2% of the patients in the training group received pCR after NAC, and this rate is higher than those reported in other studies (Houssami et al., 2012), potentially because we ruled out patients who did not undergo surgery and did not finish a complete NAC regimen. Interestingly, in the training group, multifocal/centric lesions were identified as independent predictors for the DFS of TNBC after NAC. Many studies (Duraker and Çaynak, 2014; Lang et al., 2017) have demonstrated that multifocal/centric foci exhibit more biologically aggressive behaviour than has been observed for unifocal breast cancer, and this could influence DFS and OS. Although the multifocal/centric lexicon was not included in BI-RADS, these patients should receive more attention during postoperative follow-up. While Park et al. (2018a) found that N-stage was a predictor of DFS in breast cancer, our analysis failed to support these findings, possibly due to differences in study populations. In addition, the features at MR imaging(mass vs nonmass) was not associated with DFS in multivariate analysis of variance in our study, which was consistent with the study of Tahmassebi et al. (2019).

The prognostic ability of radiomics signatures has been demonstrated in many studies. For example, Li et al. (2016) suggested that image-based radiomics features may be helpful in assessing the risk of breast cancer recurrence. Park et al. (2018b) demonstrated that Rad-scores generated from radiomics signatures based on preoperative MRI have prognostic value. In our study, we analysed preoperative MRI findings in TNBC, a special pathological type of breast cancer, and supported the notion that the Rad-score helps stratify patients, and patients from high-risk groups need more careful follow-up management.

In this study, we developed a radiomics signature-based nomogram for the individualized prediction of recurrence in patients with TNBC after NAC. The nomogram incorporates three components of a radiomics signature with six selected features, including pCR status and MR findings indicating multifocal/centric lesions, which is promising to facilitate individualized predictions and the prediction of follow-up needs in patients with poor outcomes with regard to DFS.

Our study has several limitations. First, this is a retrospective study. Second, most of the patients were examined using MR after a biopsy, which might have affected assessments. Third, we discuss only DCE images in our study, and further prospective studies should include a variety of breast MR imaging protocols, such as T2W, DWI, and DCE-MRI.

## Conclusion

In conclusion, the results of our study show that the identified Rad-score has the potential to be used as a biomarker for risk stratification for DFS in patients with TNBC after NAC. In addition, our results show that a radiomics nomogram that incorporates a radiomics signature and MRI and clinicopathological findings can be used to facilitate the individualized prediction of recurrence in patients with TNBC after NAC and surgery. This type of quantitative radiomics prognostic model of breast cancer could be useful for precision medicine and could affect patient follow-up strategies.

## Data Availability

The raw data supporting the conclusions of this article will be made available by the authors, without undue reservation.
