# Supplementary material for: A Combined Nomogram Model to Predict Disease-free Survival in Triple-Negative Breast Cancer Patients With Neoadjuvant Chemotherapy
Source: Front Genet. 2021 Nov 12;12:783513. doi: 10.3389/fgene.2021.783513 (PMC8632946; doi:10.3389/fgene.2021.783513)
Supplement: Supplementary file 1 [file Table1.DOCX]

Supplement

Table S1. Formulas for the ten new sequential features

| p indicates patient; t indicates the phase for the DCE image. |
| --- |
| $mean=\frac{1}{N_{t}}\sum_{t=1}^{N_{t}} x_{pt}$ |
| $variance=\frac{1}{N_{t}}\sum_{t=1}^{N_{t}} {{(x}_{pt}-\bar{x}_{p\cdot})}^{2}$ |
| $\mathrm{skewness}=\frac{\frac{1}{N_{t}}\sum_{t=1}^{N_{t}} {(x_{pt}-\bar{x}_{p\cdot})}^{3}}{\left( \sqrt{\frac{1}{N_{t}}\sum_{t=1}^{N_{t}} {(x_{pt}-\bar{x}_{p\cdot})}^{2}} \right)^{3}}$ |
| $\mathrm{kurtosis}=\frac{\frac{1}{N_{t}}\sum_{t=1}^{N_{t}} {(x_{pt}-\bar{x}_{p\cdot})}^{4}}{\left( \sqrt{\frac{1}{N_{t}}\sum_{t=1}^{N_{t}} {(x_{pt}-\bar{x}_{p\cdot})}^{2}} \right)^{2}}$ |
| $energy=\sum_{t=1}^{N_{t}} {x_{pt}}^{2}$ |
| $entropy=-\sum_{t=1}^{N_{g}} p_{t}{log}_{2}\left( p_{t}+\epsilon\right)$  $P_{t}$ is the first-order histogram with $N_{g}$ discrete intensity levels, where $N_{g}$ is the number of non-zero bins  $p_{t}$ is the normalized first-order histogram and is equal to $\frac{P_{t}}{N_{t}}$.  Here, ϵ is an arbitrarily small positive number (≈2.2×${10}^{-16}$). |
| Kendall's tau-b  Kendall's tau-b is a nonparametric measure of association based on the number of concordances and discordances in paired observations. In this study, Kendall's tau-b was used to measure the coordinated consistency of changing features between one patient and the remaining patients.  The consistent coefficient between two patients (p and p’) at two time points (t and t’) was calculated as follows (Equation (1)):  $I=\frac{x_{p't'}-x_{pt'}}{x_{p't}-x_{pt}} (1)$ (p=1, 2,…,$p_{t}$, p’=p+1,p+2,…,$p_{t}$ , t=1,2,…,$N_{t}$ , t’=t+1,t+2,…,$N_{t}$,)  where x is the value of one of the texture features, $N_{p}$ is the number of patients, and $N_{t}$ is the number of time points. Therefore, the total number of $N_{t}$*($N_{t}$-1)/2 -consistent coefficients were obtained for each pair of patients.  The consistent coefficients are subsequently subclassified into four groups: $I>0, I<0, I=0 and I\to\infty$. The corresponding numbers of items were: $N_{I>0},N_{I<0},N_{I=0}$ and $N_{I\to\infty}$ .  Kendall's tau-b was calculated as follows:  $\tau=\frac{N_{I>0}-N_{I<0}}{\sqrt{(N_{I>0}+N_{I<0}+N_{I=0})\times(N_{I>0}+N_{I<0}+N_{I\to\infty})}} (2)$ |
| Conservation  The conservation of patients was calculated as follows:  $r_{pp'}=\left\vert\frac{N_{t}\sum_{t=1}^{N_{t}} x_{pt}x_{p't}-\sum_{t=1}^{N_{t}} x_{pt}\sum_{t=1}^{N_{t}} x_{p't}}{\sqrt{N_{t}\sum_{t=1}^{N_{t}} x_{pt}^{2}-{(\sum_{t=1}^{N_{t}} x_{pt})}^{2}}\sqrt{N_{t}\sum_{t=1}^{N_{t}} x_{p't}^{2}-\left( \sum_{p=1}^{N_{t}} x_{p't} \right)^{2}}} \right\vert(3)$  Here, the absolute value of the Pearson correlation coefficient was used because both positive and negative values indicate that the two patients were relatively conserved. |
| Stability  Stability was defined by Equation (4):  $S_{pp'}=1-\max\left\{ {PCV}_{p},{PCV}_{p} \right\} (4)$  where ${PCV}_{p}$(or ${PCV}_{p'}$) indicated the percentage of patients whose coefficient of variation (CV) did not exceed the CV of patient $p$ (or $p'$).  ${PCV}_{p}=\frac{number of patients with CV<{CV}_{p}}{total number of patinets(N_{p})} (5)$ |
| Dispersion  Let $\mathrm{Max}_{p}=max(x_{p1},x_{p2},\ldots,x_{pT})$,$\mathrm{Min}_{p}=min(x_{p1},x_{p2},\ldots,x_{pT})$,$\mathrm{Max}_{p'}=\max\left( x_{p'1},x_{p'2},\ldots,x_{p'T} \right)$, $\mathrm{Min}_{p'}=\min\left( x_{p'1},x_{p'2},\ldots,x_{p'T} \right)$.  We assumed that $\mathrm{Max}_{p}$ was larger than $\mathrm{Max}_{p'}$, and the dispersion was therefore defined as follows:  $D_{pp'}=\left\{ \begin{aligned} 0 if {Max}_{p}\geq{Max}_{p'}\geq{Min}_{p'}\geq{Min}_{p} \\ 1-\frac{{Max}_{p}'-{Min}_{p}}{{Max}_{p}-{Min}_{p^{'}}} if {Max}_{p}\geq{Max}_{p'}\geq{Min}_{p}\geq{Min}_{p'} \\ 1 if {Max}_{p}\geq{Min}_{p}\geq{Max}_{p'}\geq{Min}_{p'} \end{aligned} \right.$ (6) |
